# A Study on Decision-Making for Improving Service Efficiency in Hospitals

**DOI:** 10.3390/healthcare12030405

**Published:** 2024-02-04

**Authors:** Su-Wen Huang, Shao-Jen Weng, Shyue-Yow Chiou, Thi-Duong Nguyen, Chih-Hao Chen, Shih-Chia Liu, Yao-Te Tsai

**Affiliations:** 1 Department of General Affairs, Taichung Veterans General Hospital, Taichung 40705, Taiwan; dale33663366@gmail.com (S.-W.H.); yow0819@vghtc.gov.tw (S.-Y.C.); 2 Department of Information Management, Chaoyang University of Technology, Taichung 41349, Taiwan; 3 Department of Industrial Engineering and Enterprise Information, Tunghai University, Taichung 40704, Taiwan; sjweng@thu.edu.tw (S.-J.W.); p8311011@gmail.com (C.-H.C.); 4 Department of Business Administration, National Chung Hsing University, Taichung 402202, Taiwan; duongyennguyen@gmail.com; 5 Department of Information Management, National Kaohsiung University of Science and Technology, Kaohsiung 82445, Taiwan

**Keywords:** intelligent dispatch, system simulation, healthcare resource allocation, service efficiency, patient safety, healthcare quality

## Abstract

The provision of efficient healthcare services is essential, driven by the increasing demand for healthcare resources and the need to optimize hospital operations. In this context, the motivation to innovate and improve services while addressing urgent concerns is critical. Hospitals face challenges in managing internal dispatch services efficiently. Outsourcing such services can alleviate the burden on hospital staff, reduce costs, and introduce professional expertise. However, the pressing motivation lies in enhancing service quality, minimizing costs, and exploring innovative approaches. With the rising demand for healthcare services, there is an immediate need to streamline hospital operations. Delays in internal transportation services can have far-reaching implications for patient care, necessitating a prompt and effective solution. Drawing upon dispatch data from a healthcare center in Taiwan, this study constructed a decision-making model to optimize the allocation of hospital service resources. Employing simulation techniques, we closely examine how hospital services are currently organized and how they work. In our research, we utilized dispatch data gathered from a healthcare center in Taichung, Taiwan, spanning from January 2020 to December 2020. Our findings underscore the potential of an intelligent dispatch strategy combined with deployment restricted to the nearest available workers. Our study demonstrates that for cases requiring urgent attention, delay rates that previously ranged from 5% to 34% can be notably reduced to a much-improved 3% to 18%. However, it is important to recognize that the realm of worker dispatch remains subject to a multifaceted array of influencing factors. It becomes evident that a comprehensive dispatching mechanism must be established as part of a broader drive to enhance the efficiency of hospital service operations.

## 1. Introduction

### 1.1. Background and Related Works

In the medical service system, several crucial elements hold the key to patient satisfaction, cost management, and overall service quality [1]. As hospitals grapple with capacity constraints and operational inefficiencies within Taiwan’s healthcare landscape, the need for significant improvements becomes increasingly evident. The challenge lies in leveraging medical resources, time, and efficiency to meet patients’ needs and enhance their satisfaction.

The healthcare system in Taiwan heavily relies on the National Health Insurance, which covers the majority of the population. This results in hospitals facing immense pressure to efficiently handle a large number of patients within limited timeframes [2]. However, these hospitals often grapple with capacity constraints and operational inefficiencies, compelling the need for significant improvements. Policymakers actively seek solutions to enhance the healthcare system, focusing on cost containment and production improvements [3]. Research has consistently shown that optimizing hospital productivity is linked to effectively managing patient flow within healthcare organizations.

Efforts to elevate medical quality have predominantly revolved around enhancing various aspects of medical performance, such as the reduction in length of stay (LOS) in emergency departments, improved appointment scheduling [4,5,6,7,8,9,10], and optimized resource allocation [11,12,13,14]. However, a crucial yet often overlooked component of hospital operations is the role of hospital porters, known as non-clinic service personnel. Service personnel are trained non-clinical healthcare workers whose duties include patient transport for testing, document service, inpatient medication delivery, and other relevant services. Although each hospital assigns different obligations and responsibilities, the general duty of service personnel is mainly focused on patient transport or goods delivery. Studies have found that service personnel usually receive on-the-job training. These dedicated professionals are responsible for facilitating the day-to-day functioning of hospitals [15]. They transport patients to various locations, including moving them from wards to X-ray rooms, and ensuring timely deliveries of specimens, medications, and patients’ records. Hospital porters are sometimes referred to as support services assistants or patient services assistants. When a service is required, a ward or department submits a request, initiating the process of job dispatch, service delivery, and confirmation, each vital for the smooth operation of hospitals [15].

Despite their critical role, service personnel have received minimal attention from hospital management and have rarely been the focus of comprehensive studies. While numerous studies on healthcare and hospital operations exist, very few delve into the impact of porter services [15,16].

Recognizing the vital role that porter services play in hospital efficiency and patient care is essential to driving improvements in the overall healthcare system. Enhancing the performance of this sector can significantly contribute to relieving the pressure on hospitals and optimizing patient experiences. However, the performance of hospital service personnel faces issues related to task unfamiliarity and working environment constraints. The most significant issues they face at work include unfamiliarity with task instructions and the working environment, which could delay task completion. Accordingly, the ability of service personnel to complete their tasks largely depends on their experience level. These challenges often lead to delays in task completion. 

Failure to deliver porter services punctually can have far-reaching consequences. Patients may miss appointments and need to reschedule, leading to delays in their treatment schedules and transportation [16]. These delays create bottlenecks in the healthcare system, resulting in longer waiting lines [17]. This issue was prominently highlighted during a study of CT scanner operations at Vancouver General Hospital. It became evident that one of the primary factors affecting the efficient use of CT scanners was delays caused by the unavailability of porters, which prevented timely patient transfers. This scenario is not unique to Vancouver General Hospital; it is a common challenge in hospitals where valuable resources like CT scanners, MRIs, or operating rooms are rendered inaccessible or underutilized due to the unavailability or delayed response of a seemingly less expensive resource—the porter [18].

The operating procedures for service personnel vary across Taiwan’s hospitals due to the different organizational models in each hospital. Some hospitals have centralized management and unified labor dispatch, whereas others use workers with fixed stations or mixed marshaling. Some utilize centralized management and unified labor dispatch, while others prefer workers with fixed stations or mixed marshaling. Yet, most dispatching operations continue to rely on traditional methods, often using telephone communication. Traditionally, manual dispatching led to inefficiencies, task allocation issues, and ad-hoc assignments. Transport services are fundamental to daily operations in hospitals, and using service personnel can maximize the workforce and reduce costs. Before decision-making systems were introduced to hospitals, dispatching and allocating tasks were manually performed. This approach failed to fully coordinate services to meet the demand of each floor or department and may lead to resource wastage due to over- or short-staffing. In addition, service personnel often had to take the initiative to determine if their support was required and sometimes had their tasks assigned on the spot by clinical staff. In such a system, hospital staff cannot understand how many tasks service personnel could complete in a day or if tasks are being completed efficiently. In addition, applying big data can improve economic and efficient effects on the hospital’s service system. However, the typical problem is the risk of cost control caused by improper workforce allocation [19]. 

### 1.2. The Research Objectives and Research Flow

The current study focuses on the need for efficient healthcare services in response to the escalating demand for healthcare resources and the need to optimize hospital operations. Acknowledging the challenges hospitals face in managing internal dispatch services, the study emphasizes the potential benefits of outsourcing to alleviate staff burdens, reduce costs, and introduce professional expertise. The driving force behind this research lies in the critical motivation to enhance service quality, minimize costs, and explore innovative approaches. With the rising demand for healthcare services, the study underscores the urgency of streamlining hospital operations, particularly in the context of internal transportation services. Delays in these services can have profound implications for patient care, necessitating prompt and practical solutions. Drawing upon dispatch data from a healthcare center in Taiwan, the study constructs a decision-making model to optimize the allocation of hospital service resources. The research uses simulation techniques to examine the current hospital service organization and operations closely. Integrating computer information systems and data-driven approaches is proposed to improve hospital service operations, addressing challenges related to cost control and workforce allocation complexities. The study aims to answer how healthcare organizations can enhance service quality, innovate services, and reduce clinical operation costs. The research constructs a simulation model for hospital service personnel operations to achieve this, proposing human resource allocation strategies for enhancement and analysis. The ultimate goal is to analyze the current operational strategy for dispatching service personnel, identify bottlenecks, and propose efficiency-improving plans based on dispatch data obtained from healthcare centers in Taichung, Taiwan, from January 2020 to December 2020.

This research is organized into key sections. The introduction sets the stage by highlighting the demand for healthcare resource optimization, focusing on internal dispatch operations. The Section 2 details the research design, utilizing simulation techniques and dispatch data from a Taichung healthcare center (January 2020 to December 2020). Section 3 showcase the positive impact of an intelligent dispatch strategy, especially in reducing delay rates for urgent cases. The Section 4 explores the complexities of worker dispatch, emphasizing the need for a comprehensive mechanism. Section 5 synthesize the findings, emphasizing the potential for improved hospital service efficiency through intelligent dispatch strategies and the ongoing need for advancements in healthcare operations.

## 2. Materials and Methods

### Simulation Modeling

Our study drew upon dispatch data collected from a healthcare center in Taichung, Taiwan, covering the period from January 2020 to December 2020. This dataset included crucial information, such as task descriptions, operation durations, task classifications, dispatch timestamps and locations, and the time taken for service personnel to reach their designated service points. According to the data analysis based on dispatch data obtained from healthcare centers, including the task arrival time, the time between the hospital establishing a task and the service centers receiving it, the time between task dispatch and task receipt, and the time between task receipt and arrival at the service location, a model is constructed (Table 1) to simulate the process for dispatching service (extremely urgent task cases). Simul8 version 30 is used to build the model.

In healthcare optimization, simulation stands out as a powerful and flexible tool, as emphasized by Valinsky [20]. It offers a dynamic platform for testing, hypothesizing, devising strategies, and shaping new healthcare designs. Notably, this method has been widely adopted to address multifaceted challenges in healthcare settings. System simulation has been instrumental in refining patient flow [21,22,23] and estimating the requisite number of intensive care unit (ICU) beds [24]. It has also played a pivotal role in scrutinizing and enhancing the allocation of resources within emergency medical systems [25]. This approach has further been deployed to curtail the costs associated with patient transport to hospitals [26] and to reduce patients’ waiting times [25]. In response to the persistent issue of long waiting times in healthcare facilities, various studies have harnessed simulation to identify the underlying causes [24,27,28,29]. Moreover, strategies have been developed to reduce outpatient waiting times [30]. Simulation has also been instrumental in assessing and comparing appointment systems [31] and forecasting emergency department crowding through real-time evaluations [32].

Our study employed a comprehensive simulation model structured as follows:

- Categorization of Services: Services were classified into three categories, encompassing patient services, fixed event services, and emergency services. The model incorporated historical statistical data to input the frequency of events during morning, evening, and night shifts.
- Human Resource Allocation Strategies: The model integrated various human resource allocation strategies tailored to floor-based personnel and mobile personnel. These mechanisms were designed to enhance the effective allocation of resources based on operational requirements.
- Task Workflow Optimization: An essential aspect of the simulation model was workflow optimization for service personnel. After completing an assignment, they were required to promptly notify the service center before proceeding to the next task. This streamlined communication facilitated a more efficient allocation of resources.

Our study leveraged real-world dispatch system data to construct a simulation model and propose resource allocation strategies to enhance service dispatch operations. The analysis of the existing dispatch strategy allowed us to identify operational bottlenecks in service personnel’s workflow. In response, we devised a plan to maximize the utilization of hospital service resources, ultimately improving overall operational efficiency within healthcare settings.

## 3. Results

### 3.1. Analysis of the Current Dispatch Strategy

It is essential to break down the process into its fundamental components to gain a deeper understanding of the current dispatch strategy employed by service centers. This deconstruction reveals a sequence of crucial steps:

1. Task Establishment: The process initiates with the hospital establishing a task, signaling the need for service personnel to execute a specific assignment.
2. Task Dispatch: The task is then dispatched to the appropriate service personnel, a process that can be handled via the dispatch system or through manual intervention.
3. Dispatch Notification: Service personnel receive a notification once the task is dispatched, acknowledging the assignment.
4. Task Execution: Following the receipt of the task, service personnel proceed to the service location, where they execute the assigned task, ensuring the seamless delivery of healthcare services.

**Figure 1:**
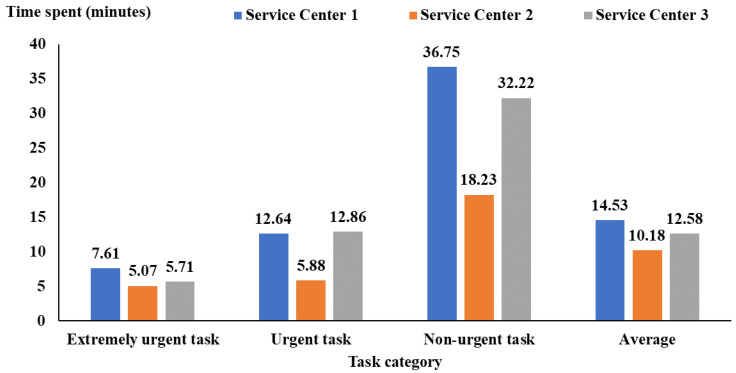
The time between the hospital establishing a task and the service centers receiving the task order.

Figure 1 visualizes a detailed analysis of the duration between the hospital’s task establishment and the service center’s receipt of the task order. This analysis spans a significant timeframe, from January 2020 to December 2020. In addition, this duration analysis is categorized into three distinct urgency levels: “extremely urgent”, “urgent”, and “non-urgent” tasks.

The findings indicated that regardless of the service category under consideration, Service Center 2 was found to have the best performance in the shortest time. In contrast, Service Center 1 experiences the most significant room for improvement, demonstrating the worst performance, and Service Center 3 falls in the middle. When we calculate the average time (95% CI) across these three categories, Service Center 1 averages 14.53 (95% CI: 14.42 to 14.64) minutes, Service Center 2 excels with an average of 10.18 (95% CI: 10.04 to 10.32) minutes, and Service Center 3 demonstrates efficiency with an average of 12.58 (95% CI: 12.35 to 12.81) minutes. This data highlights the importance of reducing the time it takes to start tasks across all service categories, as shorter times lead to better operational efficiency and quicker service.

The findings of the duration analysis provide important information. By understanding this information, we can picture a solution to significantly improve how tasks are handled in health care centers.

**Figure 2:**
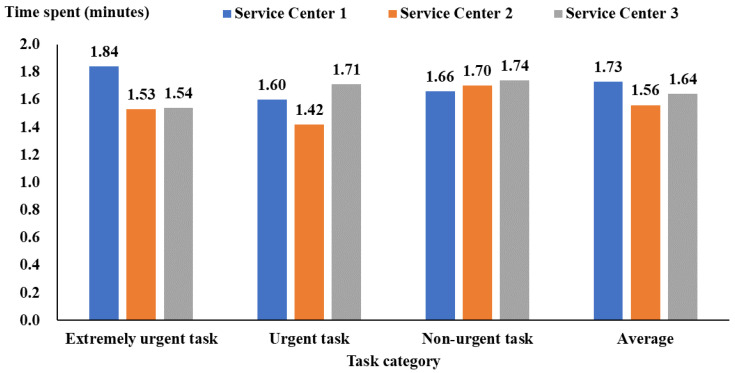
The time between task dispatch by service centers and task receipt by service personnel.

**Figure 3:**
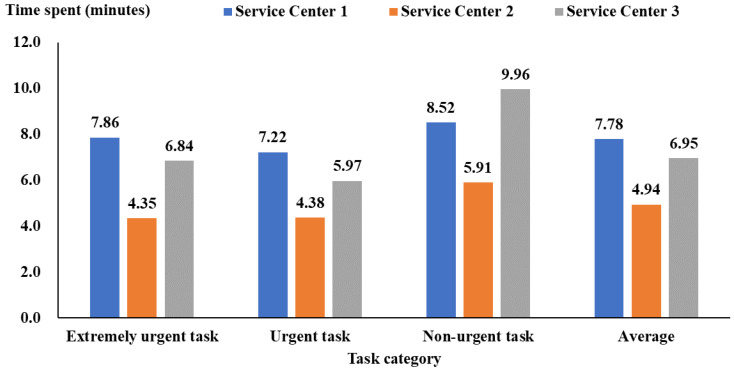
The time between task receipt by service personnel and arrival at the service location.

Figure 2 shows the duration between task dispatch by the service center and task receipt by service personnel, which was 1.73 (95% CI: 1.72 to 1.74), 1.56 (95% CI: 1.53 to 1.59), and 1.64 (95% CI: 1.60 to 1.68) minutes for Service Centers 1, 2, and 3, respectively. Service Center 2 generally had the best performance (Figure 2). Figure 3 also shows that the duration between task receipt and arrival at the service location was 4.94 (95% CI: 4.89 to 4.99) minutes for service personnel from Service Center 2, shorter than Service Centers 1 and 3 (more than 6 min).

### 3.2. Analysis of Delay Rate

Service centers employ the time it takes for a task to go from being received to reaching its intended location to gauge service delays. In the context of our study, we categorize tasks into three urgency levels. In the case of “extreme urgency”, when the service center receives the dispatch of a responsible person so assigned, the start point must be reached within 15 min. Most cases push patients for surgery, emergency examinations, or emergency medical services. Extremely urgent cases should ideally be serviced within 15 min, while urgent and non-urgent cases are expected to be attended to within 30 and 60 min, respectively.

Data collected from service centers during 2020 shows that the delay rates for urgent and highly urgent cases exhibit variability across Service Centers 1, 2, and 3. The delay rates were approximately 40.91%, 21.13%, and 31.38% for these respective service centers, and the average delay rate of the general dispatch service is 36.42%. Also, the validating results of simulating the process for dispatching service were obtained in Table 2. Table 2 shows these three service centers’ simulated means and 95% confidence intervals of the delay rates for highly urgent cases. The simulation results and historical data are similar.

Analyzing the root causes of these delays found a complex interplay of factors. Delays usually happen because it is tricky to plan when the service personnel are available, where the service is needed, and whether the service order is logistically feasible. In essence, these delays are closely linked to the time it takes to dispatch, which, in turn, links to the availability of service personnel.

To provide a clearer picture of this process, we have created a simulation model that elucidates the sequence of steps in the current dispatch operating procedures. These steps encompass the task’s establishment, its receipt by service centers, the receipt of the dispatch order by service personnel, the subsequent response to the dispatch, and the commencement and execution of the task. This detailed examination sets the stage for exploring strategies to curtail delays and enhance the efficiency of service delivery within healthcare centers.

Considering the need to lower the delay rate of extremely urgent service cases, the following strategies were simulated.

- Strategy 1: Proximity dispatch
- Strategy 2: Intelligent dispatch without delay (i.e., service centers dispatch workers immediately through the dispatch system after receiving the request)
- Strategy 3: Intelligent dispatch without delay + proximity dispatch
- Strategy 4: Intelligent dispatch within three minutes
- Strategy 5: Intelligent dispatch within three minutes + proximity dispatch

It is crucial to clarify the terminology here: “Intelligent dispatch” indicates the automated assignment of tasks to workers through the system, while “Intelligent dispatch without delay” refers to situations where the system instantly assigns tasks, with delays only occurring when suitable service personnel are unavailable.

Table 3 presents the delay rates for highly urgent cases under each strategy and for each service center, revealing a notable reduction in delay rates with the implementation of intelligent dispatch. For example, in Service Center 1, the delay rate decreased significantly from 40.91% to a mere 17.32% when employing Strategy 2, which involves immediate dispatch upon request. The combined approach of Strategy 3, incorporating both immediate intelligent dispatch and proximity dispatch, even further improved the situation, reducing the delay rate to 16.49%.

One noteworthy insight from our analysis is that the same strategy yielded distinct results across different service centers. This is evident in the outcomes of Service Center 1 and Service Center 3. For instance, by implementing Strategy 3, the delay rate in Center 1 decreased by nearly 24%, plummeting from 40.91% to just 16.49%, with an improvement rate of 59.69%. In contrast, the rate at Center 3 experienced a more significant reduction in 26.75%, decreasing from 31.38% to 4.63%, with an improvement rate of 85.24%.

These variations underscore the importance of tailoring strategies to individual service centers’ specific needs and dynamics, acknowledging that what works remarkably well for one may produce different results for another.

## 4. Discussion

In recent years, major healthcare institutions have faced significant challenges due to the COVID-19 pandemic. These challenges affect patients and have far-reaching implications for hospital operations, including the efficiency of service dispatch. Hospital service personnel are vital in supporting healthcare delivery, requiring effective communication with medical staff to ensure patient safety and quality care. Our analysis of past dispatch data and simulations of different strategies has provided valuable insights. We found that implementing intelligent dispatch, particularly when integrated with proximity-based dispatch, can significantly enhance service efficiency and reduce medical delays.

However, it is important to note that several human factors influence the real-world application of these strategies. Variables such as the volume of service orders, the coordination of medical units, and the mobilization of human resources considerably impact operational efficiency. Insufficient staffing and a high rate of unfulfilled service orders often lead to increased delays. This highlights the need for further refinement in service center dispatch mechanisms and resource allocation to prioritize patient safety.

The use of Information and Communication Technology (ICT) in the healthcare industry is increasingly important. It offers the potential to improve medical efficiency, reduce workforce-related costs, and enhance service quality. This shift towards patient-centered holistic medical care emphasizes accurate medical diagnosis and treatment and considerations like patient convenience, safety, immediacy, acceptance, comfort, and the integrity of patient services, addressing their physical, psychological, social, and spiritual needs.

In 2019, our cooperative hospital fully implemented the ‘Beacon’ real-time positioning system to cover the movements of service personnel. This system’s primary goal is to establish an intelligent dispatch system that efficiently deploys workers to the nearest locations for healthcare services. As a result, it has streamlined processes, reduced the round-trip workforce, and improved overall efficiency. Our statistical analysis demonstrates that Beacon has substantially been impacted by reducing both dispatch times and delay rates.

For example, the average dispatch time for ‘General Dispatch’ is 5 min and 23 s, while Beacon Intelligent Dispatch clocks in at just 1 min and 13 s, marking a remarkable 75.85% reduction in time. Similarly, at the start location, ‘General Dispatch’ takes 12 min and 44 s, while Beacon intelligent dispatch takes only 7 min and 17 s, resulting in a 42.8%-time improvement. Furthermore, the average delay rate for ‘General Dispatch’ is 30.3%, compared to Beacon intelligent dispatch’s 11.67%, a 61.49% decrease in delays.

Nonetheless, apart from handling extremely urgent cases intelligently assigned by Beacon, other case levels (general, urgent, and scheduling) still rely on manual dispatch by the service center’s team leader due to a limited workforce. After completing Beacon’s intelligent dispatch, the team leader often cancels completed Beacon intelligent dispatch cases due to the following reasons:

- The manual dispatch team leader considers the task type and location of the attendant, especially in cases involving multiple tasks and dispatchers, to optimize manpower usage. Simultaneously, Beacon’s intelligent dispatcher can only handle one service case at a time, which can be inefficient, leading the team leader to consider case volume and opt for manual dispatch.
- When a teleporter finishes a prior case, the team leader intends to assign them a scheduled case. However, the intelligent dispatch system sometimes interferes by automatically assigning a new task to the teleporter, resulting in a clash between manual and intelligent dispatch systems.
- Beacon’s intelligent dispatch initially follows the principle of dispatching delivery personnel for continuous inspection tasks, such as electrocardiograms, X-rays, anesthesia visits, and other services. However, when second and third tasks are in progress, the system assigns free transportation personnel tasks, creating a dilemma in task allocation.

## 5. Conclusions

In conclusion, our research illuminates the intricate dynamics of healthcare service dispatch and workforce allocation, particularly within the context of Taiwan’s evolving health insurance landscape. The integration of outsourcing as a strategic response to operational challenges underscores its potential to reduce costs in the face of increased competition and insurance benefit limitations. Our findings offer valuable insights into optimizing service dispatch and workforce allocation, emphasizing the continuous evaluation of strategic approaches in real time. The COVID-19 pandemic’s impact on hospital operations, particularly in service dispatch efficiency, is acknowledged, and our analysis of past dispatch data provides critical insights. Implementing intelligent dispatch, notably when integrated with proximity-based strategies, emerges as a potent solution to enhance service efficiency and reduce medical delays. However, challenges persist, notably influenced by human factors such as staffing issues and the coordination of service orders. The use of Information and Communication Technology (ICT) and real-time positioning systems, exemplified by the ‘Beacon’ system, significantly reduces dispatch times and delay rates. Despite these advancements, our study recognizes the limitations in the system’s capacity to handle extremely urgent cases efficiently, leading to cancellations by manual dispatch team leaders. Future research directions should explore tailored strategies for individual service centers, considering the distinct dynamics that impact the effectiveness of intelligent dispatch systems. Additionally, advancements in Information and Communication Technology should be leveraged to address human factors, further refining dispatch mechanisms for optimal healthcare service delivery. The evolving landscape of healthcare demands continuous innovation, and our study provides a foundation for future endeavors to enhance the efficiency of hospital service operations.

## Figures and Tables

**Table 1:** Simulation model inputs with actual data for extremely urgent tasks.

Model Inputs	Distributional Assumptions in Minutes *			
Task Arrival Time	Exponential Distribution with Mean 1.00			
	No Delay		Delay	
	Center	Distribution	Center	Distribution
The time between the hospital establishing a task and the service centers receiving	1	Triangular (0.00, 1.00, 2.65)	1	Triangular (10.00, 14.58, 15.04)
	2	Triangular (0.00, 2.00, 2.78)	2	Triangular (10.00, 13.13, 14.30)
	3	Triangular (0.00, 2.00, 2.68)	3	Triangular (9.00, 12.01, 12.89)
The time between task dispatch and task receipt	1	Triangular (0.00, 1.00, 1.23)	1	Triangular (1.00, 2.71, 2.78)
	2	Triangular (0.00, 1.00, 1.25)	2	Triangular (1.00, 2.56, 2.75)
	3	Triangular (0.00, 1.00, 1.02)	3	Triangular (1.00, 2.58, 2.88)
The time between task receipt and arrival at the service location	1	Triangular (0.00, 2.00, 3.32)	1	Triangular (12.00, 14.34, 14.57)
	2	Triangular (0.00, 2.00, 2.56)	2	Triangular (9.00, 10.90, 11.43)
	3	Triangular (0.00, 3.00, 3.47)	3	Triangular (11.00, 14.10, 14.85)

* Distributional assumptions minutes are set by actual data.

**Table 2:** The simulation results of dispatching service for highly urgent cases (Delay rate, %).

Service Center	Mean	95% CI	Actual Data
1	44.60	(26.81, 62.39)	40.91
2	20.30	(5.91, 34.69)	21.13
3	30.60	(14.11, 47.09)	31.38

CI: Confidence interval.

**Table 3:** Delay rates for highly urgent cases for each scenario and service center (%).

Service Center	Baseline	Strategy 1	Strategy 2	Strategy 3	Strategy 4	Strategy 5
1	40.91	43.82	17.32	16.49	36.91	36.20
2	21.13	20.32	0.00	0.00	0.56	0.56
3	31.38	30.64	4.63	4.63	18.43	18.43

## Data Availability

Data are contained within the article.
